# Levels of caspase-3 and histidine-rich glycoprotein in the embryo secretome as biomarkers of good-quality day-2 embryos and high-quality blastocysts

**DOI:** 10.1371/journal.pone.0226419

**Published:** 2019-12-19

**Authors:** Helena Kaihola, Fatma Gülen Yaldir, Therese Bohlin, Raghad Samir, Julius Hreinsson, Helena Åkerud

**Affiliations:** 1 Department of Immunology, Genetics and Pathology, Uppsala University, Uppsala, Sweden; 2 Department of Women’s and Children’s Health, Uppsala University, Uppsala, Sweden; 3 Fertility Unit, Örebro University Hospital, Örebro, Sweden; 4 Livio Fertility Centre Falun, Falun, Sweden; 5 GynHälsan Fertility Clinic, Minerva Fertility, Uppsala, Sweden; University of Florida, UNITED STATES

## Abstract

Morphological assessment at defined developmental stages is the most important method to select viable embryos for transfer and cryopreservation. Timing of different developmental stages in embryo development has been shown to correlate with its potential to develop into a blastocyst. However, improvements in pregnancy rates by using time-lapse techniques have been difficult to validate scientifically. Therefore, there is a need for new methods, preferably non-invasive methods based on metabolomics, genomics and proteomics, to improve the evaluation of embryo quality even further. The aim of this study was to investigate if different levels of caspase-3 and histidine-rich glycoprotein (HRG), secreted by the embryo into the culture media, can be used as biomarkers of embryo quality. In this study, a total of 334 samples of culture media were collected from *in vitro* fertilization (IVF) treatments at three different clinics. Protein analysis of the culture media was performed using multiplex proximity extension protein analysis to detect levels of caspase-3 and HRG in the embryo secretome. Protein levels were compared in secretome samples from high- and low-quality blastocysts and embryos that became arrested during development. Correlation between protein levels and time to morula formation was also analyzed. Furthermore, protein levels in secretomes from day-2 cultured embryos were compared on the basis of whether or not pregnancy was achieved. The results showed that caspase-3 levels were lower in secretomes from high-quality *vs*. low-quality blastocysts and those that became arrested (p ≤ 0.05 for both). In addition, higher HRG levels correlated with a shorter time to morula formation (p ≤ 0.001). Caspase-3 levels were also lower in secretomes from day-2 cultured embryos resulting in a pregnancy *vs*. those that did not (p ≤ 0.05). Furthermore, it was shown that caspase-3 might be used as a marker for predicting potential success rate after transfer of day-2 cultured embryos, where a caspase-3 cutoff level of 0.02 gave a prediction probability of 68% (p = 0.038). In conclusion, in future prediction models, levels of caspase-3 and HRG might be used as potential markers of embryo quality, and secreted caspase-3 levels could to some extent predict the outcome after transfer of day-2 cultured embryos.

## Introduction

The number of children born after assisted reproduction now exceeds eight million worldwide and the number of treatments per year is constantly increasing [1]. Although treatment regimens have been improved and methods for embryo selection have been fine-tuned and developed further, pregnancy rates are relatively stable [2].

Embryo selection is one of the key elements when trying to optimize the circumstances for optimal conditions in favor of a pregnancy. Even though research has been ongoing for decades, morphological assessment at defined developmental stages remains the most important method to select viable embryos for transfer and cryopreservation [3–5]. Timing of different stages in the development of an embryo, e.g. time to first cell division and time to morula, has been shown to correlate with its potential to develop into a blastocyst [6]. Implementation of the time-lapse technique has improved embryo monitoring, although it is unclear if it significantly aids in selection of the best embryo for transfer [7, 8], and improvements in pregnancy rates by using time-lapse techniques have been notoriously difficult to validate scientifically. Therefore, there is a need for new methods to improve the evaluation of embryo quality even further. Studies have been performed with a focus on finding non-invasive methods, based on metabolomics, genomics and proteomics [9–11] for the assessment of embryo quality [12–14]. Unfortunately, most studies have so far not been completely successful as regards prediction of pregnancy rates. For instance, near-infrared spectroscopy to detect metabolites from the embryo in culture media was considered a promising method, but was later found not to improve live-birth rates compared with selection by morphology alone [15]. There are indications that pre-implantation genetic testing for aneuploidy using spent culture media increases implantation rates among women older than 35 years of age [16]. However, this method is still at the research stage and further studies are required before it can be implemented in daily practice.

We have recently shown, in a pilot study, that levels of certain proteins, detected in media from cultured embryos, differ depending on whether or not embryos develop into blastocysts [17, 18]. For instance, levels of caspase-3 in the secretome were significantly lower in connection with high-quality *vs*. low-quality blastocysts [18]. There were also lower levels of caspase-3 in blastocyst secretomes in total compared with arrested-embryo secretomes, although this was not statistically significant, possibly reflecting the small number of embryos in that study. Lindgren and colleagues [18] showed that a shorter time to morula formation correlated significantly with the embryo developing into a blastocyst and also that lower caspase-3 levels were found in secretomes from embryos with a shorter time to morula formation, even though this was not statistically verified. Caspase-3 is known to be involved in apoptosis [19], and it has also been described as relevant in regulation of inflammation and in the development of various organs [19].

In previous studies, we have also shown that there are specific genes and proteins (e.g. histidine-rich glycoprotein [HRG] and phosphodiesterase 8B) in the embryo, placenta and in the woman’s blood that correlate with successful pregnancy [20–23]. Further, we have published results indicating that certain clusters and signaling pathways (angiogenic and inflammatory) are more relevant than others when it comes to the study of implantation and placentation–two processes essential for a successful pregnancy. Lindgren *et al*. [24] showed that HRG inhibits proliferation of endothelial cells and promotes migration and differentiation into capillary structures, which is important during angiogenesis. In addition, Juarez *et al*. [25] have shown that the anti-angiogenic function of HRG could be a result of endothelial-cell apoptosis, as measured by HRG-activated caspase-3 in HUVEC cells. HRG is known to interact with caspases via thrombospondins and cluster of differentiation 36 (CD36) to regulate angiogenesis and apoptosis [26].

The aim of this study was to investigate if different levels of caspase-3 and HRG, secreted by the embryo into culture media, can be used as biomarkers of embryo quality. To our knowledge, there are no studies concerning correlation between caspase-3 or HRG with embryo development and quality. Since the results of our previous studies were mainly obtained from small numbers of cryopreserved day-2 embryos (n = 47), thawed for research purposes only and cultured until day six, we now wanted to investigate secretomes from a larger number of embryos during *in vitro* fertilization (IVF) treatments and to compare secreted caspase-3 and HRG levels with embryo scoring and the outcome of IVF treatment.

## Materials and methods

### Ethics approval and consent to participate

All procedures performed in studies involving human participants were in accordance with the ethical standards of the institutional and/or national research committee and with the 1964 Helsinki declaration and its later amendments, or comparable ethical standards. This study was approved by the Regional Ethics Board in Uppsala (reference number 2015/284). Information about the study was given to the couples orally and in writing, and written consent documents were collected. All research methods were performed according to relevant guidelines and regulations.

### Study design

Culture media from 334 cultured embryos were collected in connection with fresh IVF treatments in collaboration with three clinics: the Livio Fertility Centre in Falun, the Fertility unit at the University Hospital in Örebro and the Reproduction Centre at Akademiska University Hospital in Uppsala, Sweden. Couples willing to donate media from their embryo cultures during IVF treatment were included consecutively, but the final analysis was performed as a case/control study.

Embryo development was monitored in an incubator for single droplet cultures and with a time-lapse camera, and the quality of the embryos was scored by embryologists at the clinic. After embryo culture, the culture media was frozen and stored separately for each embryo. The culture media would otherwise have been discarded if not donated for research. Protein analysis of the culture media was performed, with a focus on two selected proteins, caspase-3 and HRG. As controls in our study, fresh culture media were collected and also frozen prior to analysis. Patient data concerning IVF treatment outcome, as well as demographic information was noted. Not all IVF treatments succeeded after the first transfer, and as a result, some couples had a second transfer (or even more) of thawed embryos cryopreserved after the same treatment. Pregnancy was defined as a positive urinary hCG test result in gestational week four, followed by an ultrasound scan in gestational week seven to validate viability. Only viable pregnancies were included in the study. The results of the protein analysis were correlated with the outcome of the IVF treatments.

### Demographic data–enrolled patients and pregnancy rates

There were 50 couples enrolled in this study. The mean age and body mass index (BMI) of the women were 32.0 years (range 20.0–42.0 years, n = 50) and 24.2 kg/m^2^ (range 19.3–34.1, n = 48), respectively (Table 1). Of all women having transferred embryos in this study (n = 46), 25 (54.3%) became pregnant and there was no significant difference in pregnancy rates between women with transfers of embryos cultured until day two *vs*. embryos cultured until blastocyst stage (Table 2).

**Table 1:** Demographic data of the women included in this study.

	Total (n = 50)
**Age (median), years**	32.0 (20.0–42.0)
**BMI (median), kg/m**^**2**^	24.2 (19.3–34.1)

Data are presented as median (minimum–maximum). BMI: body mass index.

**Table 2:** Transfer of embryos cultured until day two or to blastocyst stage.

	Transferred embryos (n = 63)	p-value
**Number of SET/DET (n, %)**	53/5 (91.4%)	N/A
**Number of transferred day-2 cultured embryos resulting in a pregnancy/no pregnancy (n, %)**	12/16 (42.9%)	0.450
**Number of transferred blastocysts resulting in a pregnancy/no pregnancy (n, %)**	16/19 (45.7%)	0.612

Data are presented as numbers and percentages. The Chi-square test was used for statistical analysis when comparing pregnancy frequency after transfer of day-2 *vs*. blastocyst-cultured embryos. No statistically significant differences in pregnancy rates were found between the two groups (Pearson’s Chi-square test, p = 0.821). SET: single-embryo transfer; DET: double-embryo transfer.

When analyzing women becoming or not becoming pregnant (after one or more transfers), there was no significant difference in age, BMI, number of transfers, stimulation methods or reasons for infertility (Table 3).

**Table 3:** Demographic data of women becoming and not becoming pregnant.

	Not pregnant (n = 21)	Pregnant (n = 25)	p-value
**Age (median), years**	33.0 (20.0–42.0)	31.0 (25.0–40.0)	0.566
**BMI (median), kg/m**^**2**^	24.5 (19.9–33.3)	24.0 (19.3–34.1)^a^	1.000
**Number of embryo transfers carried out before pregnancy (median), n**	1 (1–2)	1 (1–4)	0.774
**Stimulation method** ^**b**^:			0.543
**Antagonist, n**	15	19	
**Agonist, n**	6	5	
**Reason for infertility:**			0.723
**Male factor, n**	5	9	
**Tubular factor, n**	3	3	
**Lesbian, n**	0	3	
**Endometriosis, n**	1	0	
**PCOS, n**	1	1	
**Anovulation, n**	1	1	
**Habitual abortion/adenomyosis, n**	1	0	
**Probably age, n**	2	1	
**Unexplained, n**	5	5	
**Not investigated**^**c**^**, n**	1	1	

Data are presented as median (minimum–maximum) for age, BMI and number of embryo transfers carried out before pregnancy. BMI: body mass index.

^a^BMI data missing in one case.

^b^Information about stimulation method is missing for one woman who became pregnant.

^c^Information about reason for infertility missing for one in each group. The Mann–Whitney *U* test was used for statistical analysis of age, BMI and number of embryo transfers carried out before pregnancy. For statistical analysis of the stimulation protocol or reason for infertility, Pearson’s Chi-square test was used.

### Human embryo culture and scoring

Oocytes were inseminated using fertilization media and transferred to single-step media after evaluation of fertilization. For culturing the embryos, the Livio Fertility Centre in Falun used SAGE 1-step^™^ (Origio AS, Denmark), the Reproduction Centre at Akademiska University Hospital in Uppsala used either SAGE 1-step^™^ or G-1 PLUS^™^ (Vitrolife® AB, Sweden) and The Fertility unit at the University Hospital in Örebro used G-TL^™^ (Vitrolife® AB, Sweden). Only zygotes with two pronuclei were selected for further culture.

In all the laboratories, the embryos were cultured in an EmbryoScope® (ESD) in separate culture wells (Vitrolife® AB, Sweden) at 37°C and at 6% CO_2_/5–6% O_2_. The EmbryoScope® has a time-lapse camera that takes pictures every 10–15 minutes at different focal levels. The picture sequences were saved and morphokinetics, i.e. the time to different embryonic developmental stages, was documented. Development and quality of the embryos were scored by embryologists according to routine methods in the IVF laboratory. After culturing, embryos were either transferred to the woman, cryopreserved for future treatments by the couple, or discarded. The embryos were cryopreserved according to standard operating procedures at each clinic.

In those cases where the woman had not become pregnant and had stored cryopreserved embryos, further transfer was performed. These embryos were thawed, but no media were collected from culture after thawing.

Using time-lapse sequences generated by the EmbryoScope®, embryo quality was evaluated by embryologists using standard morphological criteria for cleavage-stage embryos and blastocysts, according to the Istanbul consensus workshop on embryo assessment [5]. The scoring system for cleavage-stage embryos is based on stage-specific cell number and size, as well as on any abnormal features such as fragmentation, multi-nuclei or irregular cell divisions. For blastocysts, the highest grade of expansion is grade four and corresponds to a completely hatched blastocyst. Grade-one blastocysts have just started formation of the blastocoel. The trophectoderm and the inner cell mass are also scored, where grade one reflects a trophectoderm with many cells forming a cohesive epithelium and the inner cell mass is prominent and clearly perceived with many cells compacted and tightly adhering together. When dividing blastocysts into high and low quality, high-quality blastocysts had expanded with a fair trophectoderm and inner cell mass (at least score 322 according to the Istanbul consensus workshop) and low-quality blastocysts had not yet expanded (score 2 or less), with poor trophectoderm and inner cell mass (score 3 for both).

The decision to transfer day-2 cultured embryos or embryos cultured to blastocyst stage was based on clinical indications as well as standard operating protocols in the IVF laboratories. After the embryos had been cultured in the EmbryoScope®, scored and allocated for transfer or cryopreservation (or discarded) according to embryo quality, the culture media were collected (approximately 15 μL of 25 μL starting volume) in individual vials for each embryo and stored at -70°C or in liquid nitrogen until further analysis. Culture media were collected from all wells in one slide at the same time, directly after the last embryo had been transferred from the slide.

### Protein analysis via multiplex proximity extension assays

Protein analysis of the embryo culture media was performed using multiplex proximity extension assays (PEAs) [27, 28]. The assays were developed and the samples analyzed at the Single-Cell Proteomics Facility at the Science for Life Laboratory, Uppsala University, Sweden.

In proximity extension assays, proteins are probed using a homogeneous affinity-based technique that targets proteins using pairs of antibodies conjugated with oligonucleotides whose free 3’ ends are pairwise complementary. When a cognate antibody pair binds a target protein, the attached oligonucleotides are brought into proximity and can be extended by polymerization to create an amplifiable DNA reporter molecule, which is subsequently quantified by high-throughput real-time PCR. The use of pairwise protein detection ensures sandwich immunoassay-quality protein detection. A multiplex readout is achieved by decoding extension-generated DNA reporters by real-time PCR using primer pairs specific for cognate pairs of antibody conjugates. The readout provides relative protein quantification.

In this study we analyzed the levels of caspase-3 and HRG in 2 μL of culture media. The antibodies used to set up the PE assays include goat anti-human caspase-3 (AF-605-NA) and goat anti-human HRG (AF1869). All the antibodies were polyclonal and were purchased from R&D Systems Inc. (United Kingdom).

Each sample analysis plate was run with three negative assay controls (assay buffer with no antigen), three inter-plate assay controls (pooled culture media from cultured embryos) and three background controls for each culture medium (fresh culture media of G-1^™^ PLUS, G-TL^™^ and SAGE 1-Step^™^). For each sample, there was one goat IgG extension control probe that acts as a technical positive control.

The platform provides signal over background data on a Log2 scale, where a high value corresponds to a high protein concentration. For each protein, the background values were calculated by taking the mean of the negative controls + three standard deviations of the mean. Any signals detected below the background values were taken to be not detected. The relative levels of eight human proteins were measured in the 334 samples of embryo culture media and in the three background controls for each medium.

### Statistical analyses

For the statistical analysis, embryos were divided into several groups depending on how long they were cultured, what developmental stages they reached or if they were arrested, i.e. those that did not develop into blastocysts. In addition, within the blastocyst group, blastocysts of high quality were compared with blastocysts of low quality.

For statistical comparison of data, including demographic data and protein levels in the culture media, Pearson’s Chi-square test and the Mann–Whitney *U* test were used, since the data were not normally distributed. The data are presented as median and range (minimum–maximum). Receiver-operating characteristic (ROC) curves were constructed to assess predictive ability concerning the possibility of becoming pregnant at arbitrarily chosen caspase-3 levels. Sensitivity and specificity were calculated at the optimal cut-off time point chosen. Correlation between HRG and caspase-3 protein levels was calculated using Spearman’s rank correlation analysis, where the obtained coefficient is a measurement of the strength of the correlation between HRG and caspase-3 levels. A p-value of ≤ 0.05 was considered statistically significant. All statistical analyses were performed using the Statistical Package for the Social Sciences v. 24.0 for Windows (IBM-SPSS Inc., Chicago, IL, USA).

## Results

### Protein levels

**Fig 1:**
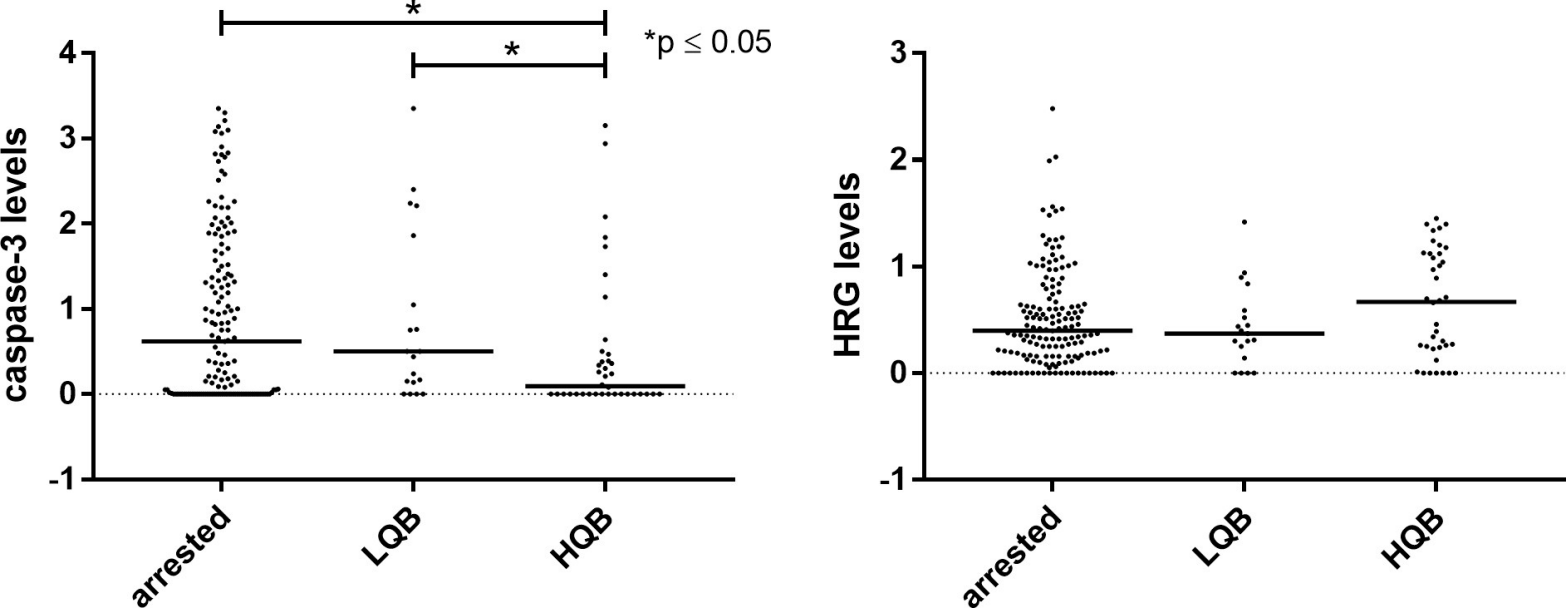
Dot plot of caspase-3 and HRG levels in secretomes from embryos cultured to blastocyst stage. Embryos reaching blastocyst stage were divided into two groups depending on blastocyst quality, i.e. low-quality blastocysts (LQB) and high-quality blastocysts (HQB). The figure shows the relative levels of caspase-3 and HRG in the embryo secretome. The Mann–Whitney *U* test was used for statistical analysis.

Protein levels in culture media were analyzed and comparisons were performed after dividing the embryos into two groups: day-2 cultured embryos and blastocyst cultured embryos. The blastocysts were furthermore divided into low-quality and high-quality blastocysts. The secretomes from high- and low-quality blastocysts were compared with those from embryos that were arrested before blastocyst stage (Fig 1), and we also compared low- *vs*. high-quality blastocysts (Fig 1). Levels of caspase-3 were significantly lower in secretomes from high-quality blastocysts *vs*. embryos that became arrested, and low-quality blastocysts (p ≤ 0.05 for both; Fig 1). No significant difference in HRG levels was found between the groups.

Even though there was no significant difference in age between women becoming pregnant and those who did not (Table 3), the effect of maternal age on the secreted levels of caspase-3 and HRG was investigated by performing a correlation analysis. There was a correlation between maternal age and secreted caspase-3 levels in all cultured embryos (n = 334) and in all transferred embryos (n = 63) (S1 Fig). There was also a significant correlation between maternal age and caspase-3 levels in the secretome when analyzing transferred blastocysts (n = 35) (S2 Fig). No significant correlations were found between maternal age and caspase-3 levels in secretomes from transferred day-2 cultured embryos (S2 Fig). Neither was there any correlation with secreted HRG levels, regardless of embryo grouping (S1 and S2 Figs).

### Correlation between protein levels and time to morula formation and cavitation

**Fig 2:**
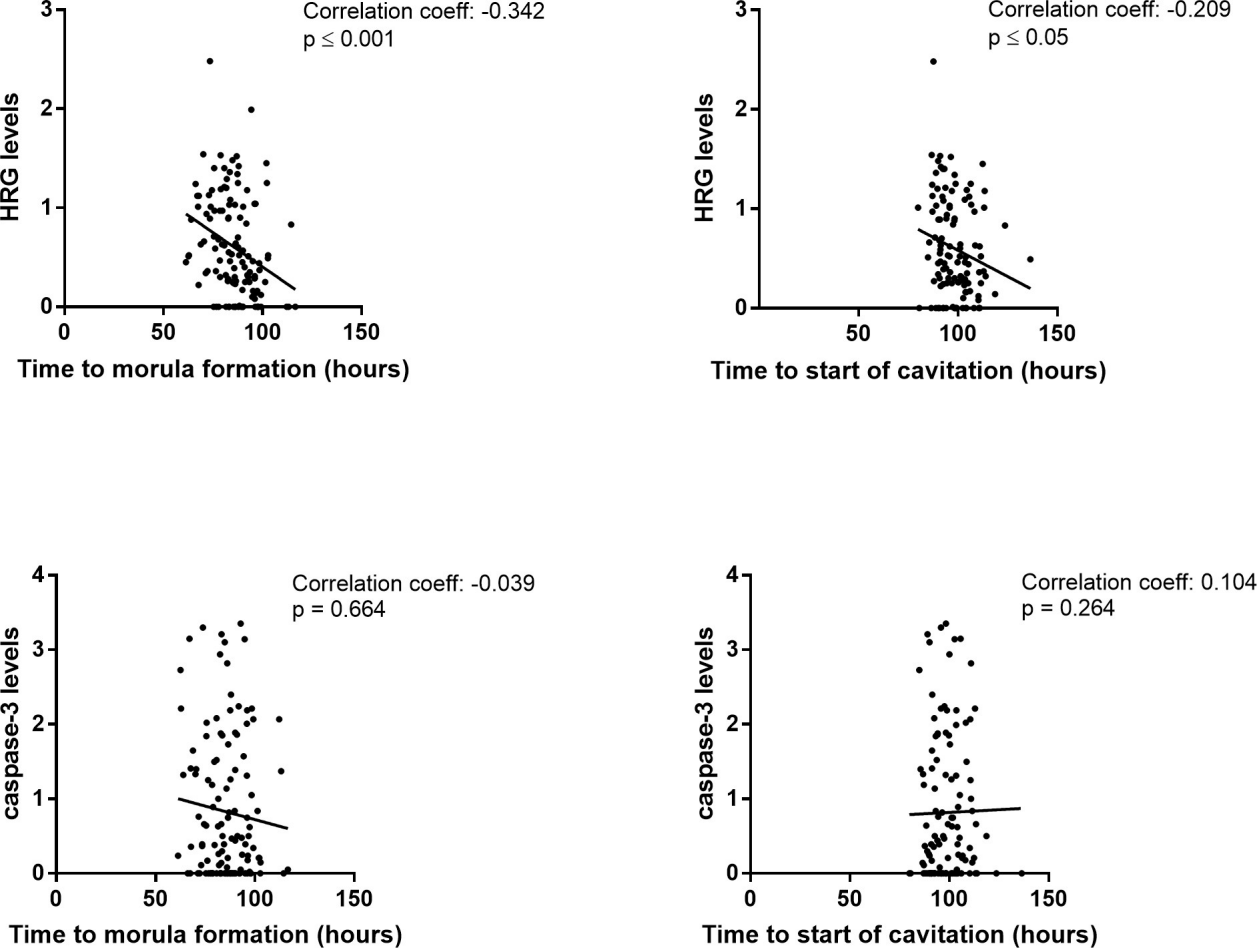
Correlation between HRG and caspase-3 levels and time to morula formation and cavitation. The scatter plot shows levels of HRG and caspase-3 measured in secretomes from all embryos reaching morula formation or start of cavitation (n = 124 and n = 117, respectively). The correlation coefficient and its significance were calculated using Spearman’s rank correlation.

When analyzing the correlation between protein levels in the secretome and time to morula formation and start of cavitation in all embryos, Spearman’s correlation analysis showed a significant negative correlation between HRG levels and time to morula formation, as well as time to start of cavitation (p ≤ 0.001 and p ≤ 0.05, respectively; Fig 2). There was no significant correlation between levels of caspase-3 and time to morula formation or start of cavitation (Fig 2).

### Protein levels and IVF treatment outcome

**Fig 3:**
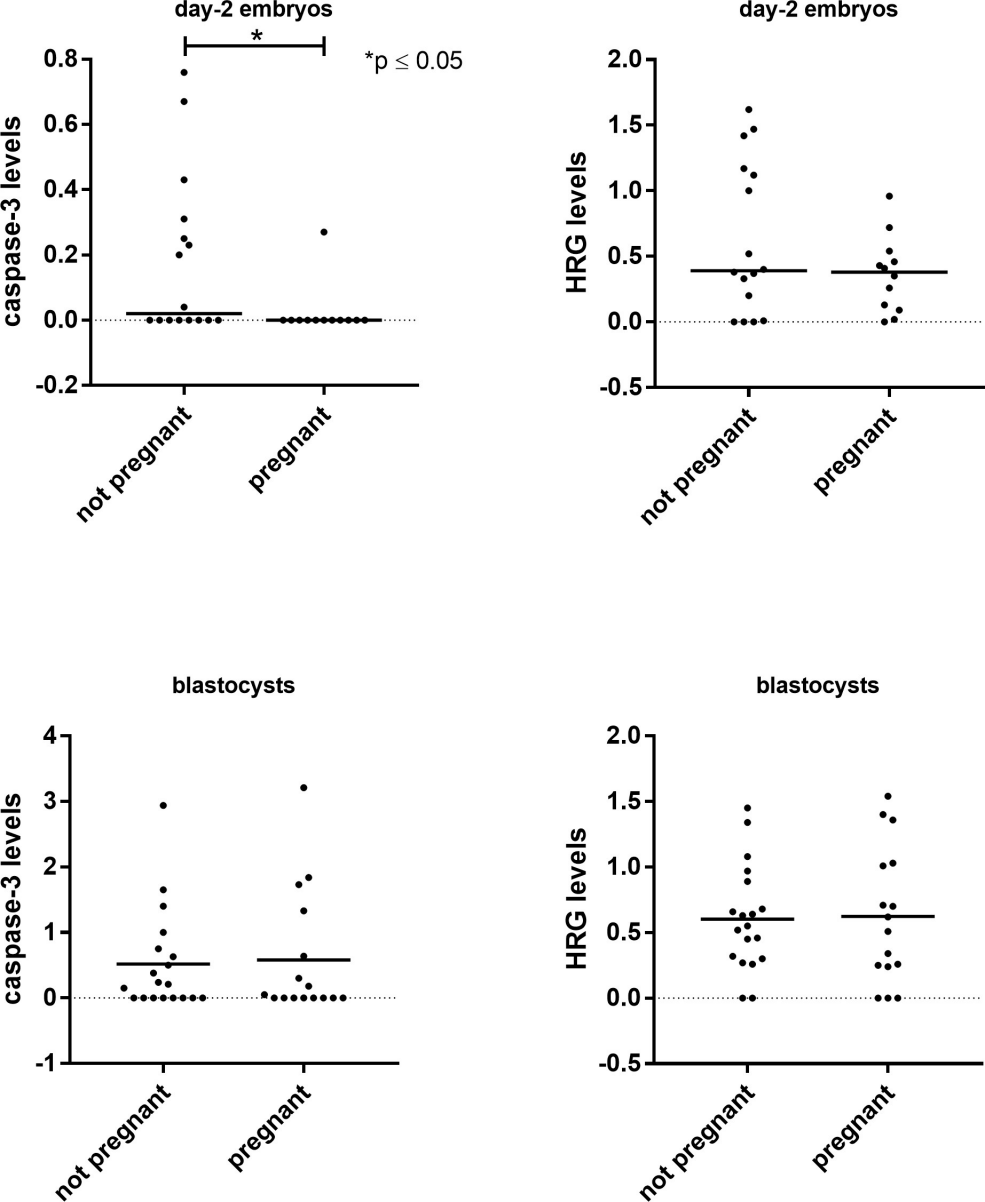
Secreted caspase-3 and HRG levels when comparing women becoming or not becoming pregnant. The dot plot shows the relative levels of caspase-3 and HRG in secretomes from transferred day-2 embryos and blastocysts. The Mann–Whitney *U* test was used for statistical analysis.

Since the quality of day-2 cultured embryos is difficult to assess, their potential quality was validated in terms of IVF treatment leading to pregnancy. In day-2 cultured embryos, secreted caspase-3 levels were significantly lower in connection with embryos resulting in a pregnancy *vs*. those that did not (p ≤ 0.05; Fig 3). No significant differences in HRG levels were found between the groups. The same analysis was also carried out for blastocysts, but no significant differences were found (Fig 3).

When analyzing caspase-3 and HRG levels in the secretomes from all transferred embryos and comparing the IVF treatment outcome, there were no significant differences between the groups (Table 4).

**Table 4:** Caspase-3 and HRG levels in secretomes from all transferred embryos resulting and not resulting in a pregnancy.

	Not pregnant (n = 35)	Pregnant (n = 28)	p-value
**Caspase-3, median (range)**	0.15 (0.00–2.94)	0.00 (0.00–3.21)	0.153
**HRG, median (range)**	0.52 (0.00–1.62)	0.42 (0.00–1.54)	0.410

Data are presented as median (minimum–maximum). The Mann–Whitney *U* test was used for statistical analysis.

**Fig 4:**
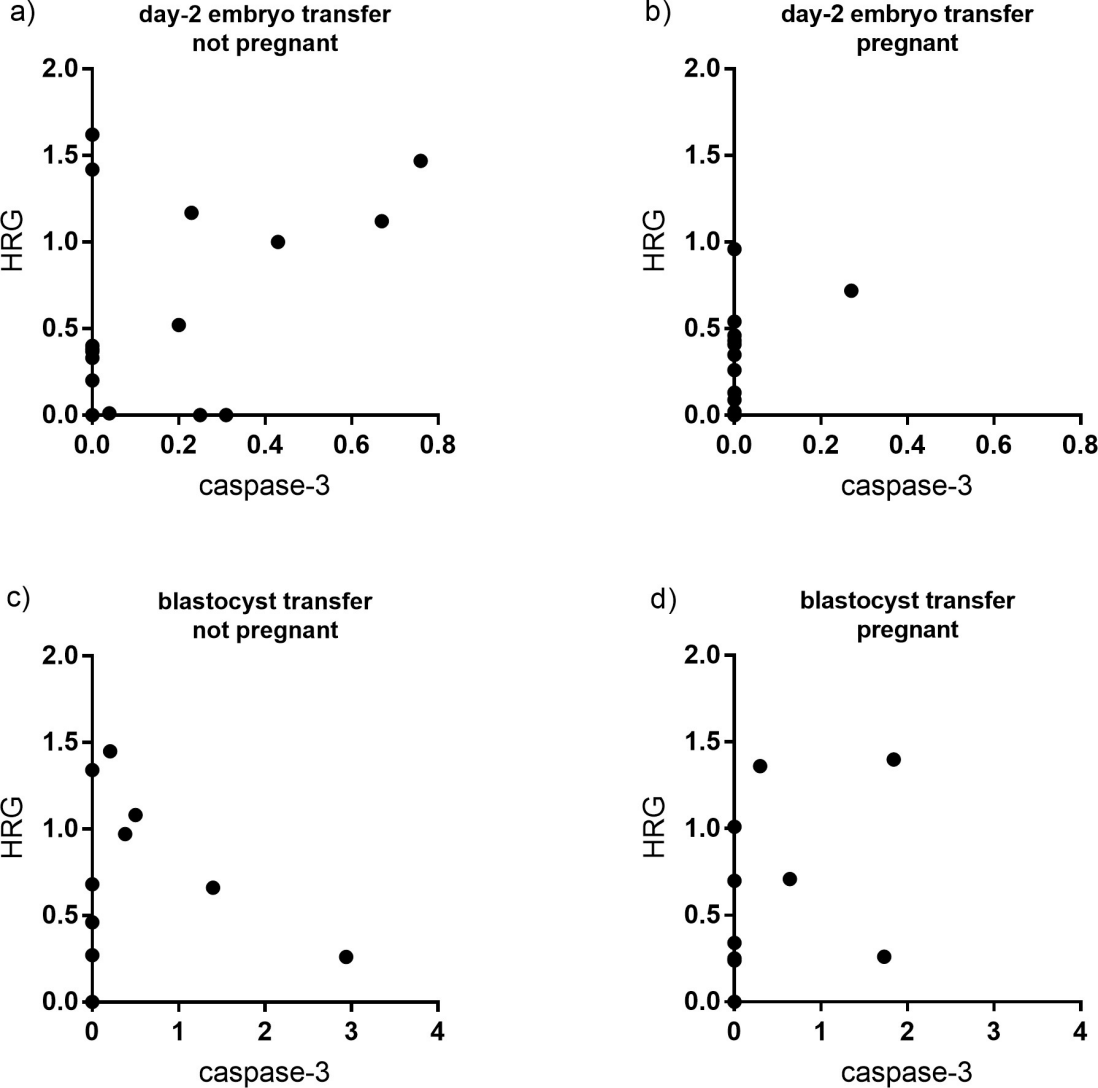
Caspase-3 and HRG levels in secretomes from embryos resulting and not resulting in a pregnancy. The scatter plot shows the relative levels of caspase-3 and HRG in culture media from each transferred day-2 cultured embryo and high-quality blastocyst resulting in and not resulting in a pregnancy. a) Day-2 cultured embryos not resulting in a pregnancy. b) Day-2 cultured embryos resulting in a pregnancy. c) High-quality blastocysts not resulting in a pregnancy. d) High-quality blastocysts resulting in a pregnancy.

To show the levels of HRG and caspase-3 in each embryo secretome, when looking at transfers resulting in a pregnancy, a scatter plot was created (Fig 4). The number of embryos with no detected caspase-3 levels in their secretome was higher among those resulting in a pregnancy (Fig 4B and 4D) *vs*. those that did not (Fig 4A and 4C). This was especially significant in the secretomes from transferred day-2 cultured embryos. Regarding high-quality blastocysts, analysis was not possible because of the low number.

Depending on the culture medium used, when looking at transferred day-2 cultured embryos, a significant difference in caspase-3 levels was found between embryos resulting in pregnancy and those that did not, but only in secretomes from embryos cultured in G-TL (S1 Table). When looking at blastocyst-cultured embryos in the same manner, there was a significant difference between high-quality blastocysts cultured in G-TL medium *vs*. low-quality blastocysts and those that became arrested (S2 Table). There was also a significant difference in secreted HRG levels between high-quality blastocysts *vs*. low-quality blastocysts and those that became arrested, when cultured in SAGE-1 medium (S2 Table). There were no significant differences in caspase-3 or HRG levels in secretomes from transferred blastocysts according to the culture medium used, when comparing IVF treatment outcome, measured as pregnancy or no pregnancy (S3 Table).

**Fig 5:**
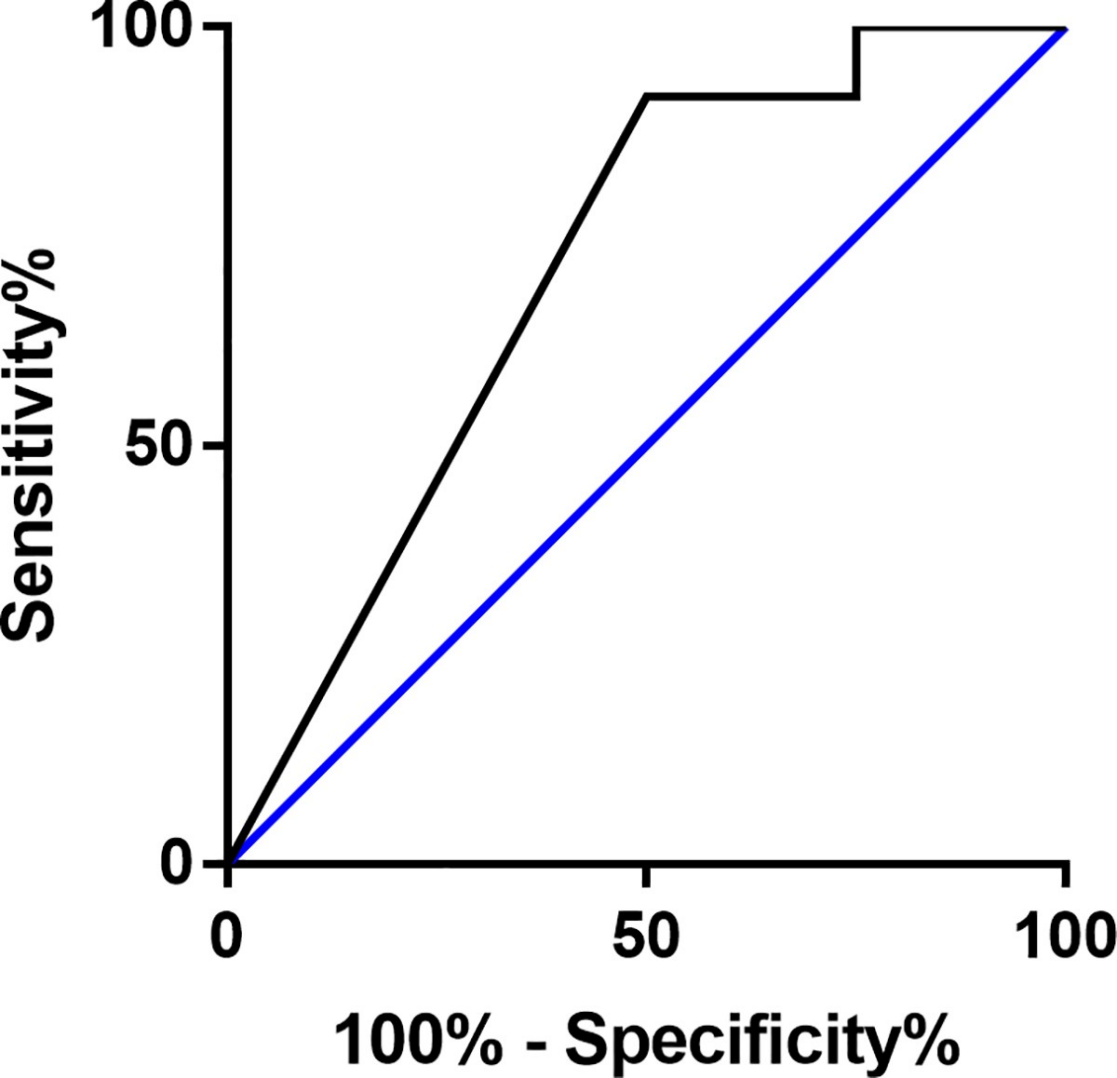
Receiver-operating characteristic curve for caspase-3 levels in secretomes from embryos cultured to day 2. The curve is based on whether or not the women become pregnant after embryo transfer. The area under the curve is 0.708. The cutoff value of caspase-3 was set to 0.02.

To analyze the secreted levels of caspase-3 in relation to predicting the possibility of becoming pregnant after transfer of day-2 cultured embryos, a receiver-operating characteristic (ROC) curve was created. At a value of 0.02, the area under the curve was 0.708, the sensitivity 92% and the specificity 50%, with an accuracy of 71% for predicting a pregnancy (Fig 5). Regression analysis was performed to evaluate if secreted caspase-3 levels could be used to predict the chance of a pregnancy after transfer of day-2 cultured embryos, using the cutoff level of 0.02 based on the ROC curve. The probability of predicting a pregnancy based on this cutoff value of caspase-3 levels was 68%, with a significance of p = 0.038 (Table 5).

**Table 5:** Binary logistic regression analysis for predicting pregnancy using a cutoff value of 0.02 for caspase-3 levels.

Coefficient	β	Std Deviation (S.E.)	Wald	df	Significance (p)	e^β^
**Caspase-3 cutoff level 0.02**	-2.398	1.158	4.288	1	**0.038**	0.091
**Constant**	2.716	1.410	3.710	1	0.054	15.125
	**Observed**	**Predicted pregnancy**		**% correct**		
		**no**	**yes**			
**Pregnant after transfer**	no	8	8	50.0		
	yes	1	11	91.7		
**Overall % correct**				**67.9**		

Relative levels of caspase-3 were detected in culture media from embryos cultured until day 2. The cutoff value was based on the results from the receiver-operating characteristics curve (Fig 5). The probability of predicting pregnancy at a caspase-3 cutoff level of 0.02 was 67.9% (p ≤ 0.05).

A ROC curve was also created for predicting a negative outcome after day-2 cultured embryo transfer. At the cutoff value for caspase-3 of 0.02, the area under the curve was 0.708, with a sensitivity of 50%, a specificity of 92% and an accuracy of 71%. Regression analysis revealed that the probability of predicting a negative outcome after transfer based on this caspase-3 cutoff value was 60%, but it was not significant (p = 0.081).

## Discussion

Even though methods for choosing the best IVF-embryo for transfer after culturing have been developed in recent years, there is still a need for more optimized selection models to improve the success rate. Today, embryo selection is mainly based on morphokinetics, but efforts are being made to find secreted metabolites and proteins that reflect the quality of the embryo. In this study, we have shown that the levels of caspase-3 and HRG in secretomes from cultured embryos correlate with embryo and blastocyst quality, as well as with IVF outcome.

Levels of secreted caspase-3 were significantly lower in embryos that reached each developmental stage at defined times according to the Istanbul consensus [5], as regards both high-quality blastocysts and day-2 cultured embryos resulting in pregnancy. Caspase-3 is a protein involved in apoptosis (programmed cell death), which is a highly regulated process involving several proteins [19]. Caspases consist of a family of conserved cysteine proteases divided into two groups: initiators and executioners [29]. Caspase-3 belongs to the group of executioners and has more than 50 known substrates, of which some are involved in DNA fragmentation [30, 31]. Oocytes and pre-implantation embryos at all stages have been found to express caspase-3, even if the embryos have less than 20% fragmentation [32]. The role of caspase-3 in apoptosis corresponds well with our findings, since the levels were higher in secretomes from embryos of poor quality and in those that became arrested during development. Embryos of poor quality often display high levels of DNA fragmentation and the reason for embryos to become arrested during development could possibly be a result of apoptosis or malfunctions in cell division [33, 34].

Levels of HRG were higher in culture media from embryos with relatively short times to morula formation, which is an important step in blastocyst development [6]. Nordqvist *et al*. [20] showed that HRG protein could be detected in the cytoplasm of human pre-implantation embryos regardless of developmental stage, even though it was a small study. They reported that higher developmental stages correlated with higher expression of HRG in the nuclei. This corresponds well with our findings, in that more highly developed embryos express higher levels of HRG, even though this did not reach statistical significance in our study.

The apoptotic effect of caspase-3 can be inhibited by HRG via interactions with thrombospondins [35]. Thrombospondins bind to CD36 and thereby initiate a cascade of events, ending in activation of caspases to initiate apoptosis. Binding of HRG to thrombospondins inhibits the binding of CD36 and consequent caspase-induced apoptosis [36, 37]. These findings correlate well with the fact that the more highly developed embryos in our study displayed both higher HRG levels and lower caspase-3 levels in the culture media than the ones that became arrested during development, even though this was not statistically significant as regards HRG.

The significant effect of maternal age on the secreted levels of caspase-3 in the embryo secretome is not surprising. It is well known that there is a positive correlation between higher maternal age and poor quality of the oocyte. However, our results are difficult to interpret, since there was a difference in the number of cultured embryos for each woman, resulting in skewness in the data together with possible effects of other demographic factors affecting caspase-3 and HRG levels. The correlation between maternal age and levels of caspase-3 in the culture media from transferred blastocysts is perhaps not unexpected, since transferred blastocysts were selected on the basis of their morphokinetic quality. The fact that there was no significant difference in age or caspase-3 levels when comparing women who became pregnant and those who did not after blastocyst transfer, indicates that the correlation found between these two parameters cannot by itself predict blastocyst quality or the outcome of IVF treatment.

When comparing pregnancy rates in connection with transfer of day-2 embryos and blastocysts, one might have expected higher pregnancy rates among women with blastocyst transfer. However, in our study success rates were similar; 42.9% and 45.7%, respectively. The reason for the high pregnancy rate after transfer of day-2 cultured embryos might be explained by the standard operating procedures used in the IVF laboratories and the careful selection of embryos made by the embryologists. The selection methods are based on morphology, timing to development, levels of fragmentation and other criteria commonly used in IVF laboratories.

There are many studies, in both humans and other species, focusing on finding non-invasive methods to detect biological markers for predicting the outcome of IVF treatment. Studying the proteome and metabolome in the embryo secretome has rendered many promising biological markers (reviewed in [11, 13, 14]). However, so far, none of these has turned out to be useful in an IVF laboratory, but there are still many markers that have not yet been tried in a clinical setup. The detection of micro-RNA (miRNA) in spent culture media has recently attracted interest. In one study, in bovines, 114 known and 180 novel miRNAs were detected in culture media, of which miR-30c was considered a novel potential biomarker, since it is involved in apoptosis and thereby also in poor embryo development [38]. Capalbo and colleagues [12] studied spent culture media from human embryos and found that miRNA profiling can be useful in connection with blastocysts, since secreted miRNA matches the miRNAs in trophectoderm cells. Interestingly, in another study, miR-20a and miR-30c were expressed in higher concentrations in culture media from implanted blastocysts compared with non-implanted blastocysts. However, the usefulness of miRNA profiling in spent culture media is still not fully elucidated [39].

Market Velker and colleagues [40] have shown that genomic imprinting in mouse embryos during *in vitro* culture differs depending on the developmental rate of the embryo. Some embryos displayed similar genomic imprinting as embryos developed *in vivo*, making this method possible for predicting the outcome after IVF treatment. In humans, some studies have suggested a correlation between IVF treatment and increased imprinting errors [41]. Rare imprinting errors leading to genetic disorders such as Beckwith–Wiedermann syndrome, Prader–Willi syndrome and Silver–Russel syndrome have been confirmed by Hattori and colleagues [42]. This is probably due to the lack of methyl donors in the culture media [43, 44]. Therefore, analysis of DNA methylation in human embryos would be helpful in choosing the best embryo for transfer. This is generally an invasive method, however, but if it could be analyzed using spent culture media, as in other forms of genetic testing [16], it would be a useful prediction model.

A predictive ability of 68% for caspase-3 might seem quite weak. However, it could perhaps be increased if used together with other parameters, such as morphokinetics or other possible proteins or metabolites. There are a few studies showing that when combining different parameters, the possibility to predict IVF treatment outcome increases. Dominguez and colleagues [45] combined morphokinetics and biochemical analysis for embryo selection and found that embryos with detected IL-6 levels and a second cell cycle duration of 5–12 hours had higher implantation rates than embryos selected by morphokinetics alone. Also, one study has shown a correlation between mitochondrial DNA content in the culture media and the embryo fragmentation rate [46]. Even though we analyzed the correlations between capsase-3 and HRG levels with time to morula formation and start of cavitation, we did not look into morphokinetic details, as did Dominguez and colleagues. It would be interesting, however, to combine several parameters to build an algorithm that could predict the outcome of IVF treatment, e.g. to combine caspase-3 levels in the secretome with detailed studies of embryo development (as in the study by Dominguez), in combination with age, BMI and possible biological markers studied in other investigations (reviewed in [47]).

Our finding of lower levels of caspase-3 in secretomes from day-2 cultured embryos resulting in pregnancy is very intriguing. In a meta-analysis by Busnelli and colleagues [48], a higher frequency of monozygotic twinning after blastocyst transfer was found when compared with cleavage-stage embryo transfer. Multiple pregnancy is a risk for both the fetus and the mother [49]. Therefore, transfer of cleavage-stage embryos would be preferred to avoid monozygotic twinning and a risky pregnancy. Hence, if it would be possible to make an early prediction, i.e. at cleavage-stage, of which embryo will result in a pregnancy, based, for example, on secreted levels of caspase-3, the chances of a higher pregnancy rate (including safe pregnancy) after IVF treatment would increase.

We also performed statistical calculations regarding the negative predictive value of caspase-3. However, we found no significant results. This could have been a result of the low number of day-2 cultured embryo transfers, and it might have been possible to obtain a figure had the sample size been larger. Again, the possibility of calculating true positive predictive values does not necessarily correlate to the possibility of calculating true negative predictive values. If statistical significance for a negative predictive value is not reached, this means that there could be, in our case, a possibility for embryos with caspase-3 values higher than the cutoff value to result in a pregnancy.

All culture media collected were from fresh cultures, not from frozen and thawed embryos. This mimics how it would be if HRG and caspase-3 were to be used in a prediction model in practice in an IVF laboratory, especially if the laboratory uses one-step media. When embryos have been cultured in an IVF laboratory, embryologists decide which embryo will be used for transfer, or freezing for future use. Therefore, measuring protein levels in the secretome at this stage would facilitate the decision concerning which embryo to choose. One could argue that the levels detected in our study are levels that accumulated during culture, and thereby do not reflect the levels at certain developmental stages, or that our findings may not be relevant in laboratories culturing embryos in sequential media. However, today, many IVF laboratories use one-step media and culture the embryos in incubators equipped with time-lapse cameras, meaning that taking samples from the culture media for analysis would preferably be done when the culture is finalized, leaving the embryos untouched until deciding which one should be chosen for transfer.

Even though the multiplex proximity extension protein assay is a promising technique for analyzing several proteins in the same droplet, the analysis takes several days to perform. This means that the embryos need to be frozen until the analysis is finalized. Today, to our knowledge, there is no available technology to detect small amounts of proteins in a small volume like embryo culture media within a few hours. However, the technology exists for blood samples, where antibody-based methods are used for quick detection of, for example, C-reactive protein (CRP), often within 10 minutes. However, this analysis method requires more than a few microliters of sample. If it would be possible to develop this technology to include detection of low amounts of proteins in small volumes of culture media, it would presumably be possible to choose the best embryo as early as at two days of culture, thereby also minimizing the risk of monozygotic twinning after IVF treatment.

One limitation in this study is that the participating IVF clinics used different culture media. We have compensated for this by subtracting the median levels of caspase-3 and HRG in the culture media themselves (meaning not exposed to embryos) from the detected levels in the media from cultured embryos. In addition, the caspase-3 and HRG levels detected in the secretomes from blastocysts were compared with the levels from embryos that became arrested before blastocyst stage. The group of arrested embryos displayed great diversity of developmental stages and embryo quality. Despite this, we found significant differences in secreted caspase-3 levels between the blastocysts and the group of arrested embryos. The fact that we found significant differences even though three different clinics were involved, could also be a positive aspect of this study, meaning that the results were significant regardless of the culture conditions used. Another limitation might be that the number of included couples was only 50 and the numbers of transferred embryos and pregnancies only 63 and 28, respectively. However, the number of culture medium samples in total (n = 334) should be sufficient for statistical analysis and we found significant results despite the low number of positive outcomes after IVF treatments.

When analyzing caspase-3 and HRG levels (and taking the different culture media into account) we found significant differences both when looking at embryo quality, and also as regards caspase-3 levels when analyzing the outcome in connection with day-2 cultured embryos. However, in some cases the groups became very small when dividing the transferred embryos into the various groups, therefore making it difficult to interpret if the culture media had had an impact on secreted caspase-3 and HRG levels, or the outcome of IVF treatment. In addition, even though there was an effect of the culture media on the quality of the blastocyst-cultured embryos, it had no effect on the overall outcome of IVF treatment, since the pregnancy rate was approximately the same regardless of the culture medium used. However, the groups were very small and this aspect needs to be studied further.

Our results indicate that the levels of secreted caspase-3 and HRG are potential markers of embryo quality. Levels of caspase-3 in the secretome could also to some extent predict IVF outcome after transfer of day-2 cultured embryos. In the future, it would be interesting to assess if this information could be used in daily routines within IVF treatment and if it could be part of the decision-making process concerning which embryo to transfer to the woman, which in turn could lead to an increased success rate in IVF treatment and favorable development of the child. Further studies are needed to elucidate the use of these markers in this context.

## Supporting information

S1 Fig Scatter plot showing the correlation between maternal age vs. caspase-3 and HRG levels in the embryo secretome. The figure shows the correlation between age *vs*. caspase-3 and HRG levels in the secretome from all cultured embryos (n = 334) and from all transferred embryos (n = 63). The correlation coefficient and significance were calculated using Spearman’s rank correlation.
(TIF)

S2 Fig Scatter plot showing the correlation between maternal age vs. caspase-3 and HRG levels in the embryo secretome. The figure shows the correlation between age *vs*. caspase-3 and HRG levels in the secretome from transferred day-2 cultured embryos (n = 28) and blastocysts (n = 35). The correlation coefficient and significance were calculated using Spearman’s rank correlation.
(TIF)

S1 Table Caspase-3 and HRG levels in secretomes from transferred day-2 cultured embryos. (DOCX)

S2 Table Caspase-3 and HRG levels in secretomes from embryos cultured to blastocyst stage. (DOCX)

S3 Table Caspase-3 and HRG levels in the secretomes from transferred blastocysts. (DOCX)
