# Supplementary figures and images for: Levels of caspase-3 and histidine-rich glycoprotein in the embryo secretome as biomarkers of good-quality day-2 embryos and high-quality blastocysts
Source: PLoS One. 2019 Dec 19;14(12):e0226419. doi: 10.1371/journal.pone.0226419 (PMC6922338; doi:10.1371/journal.pone.0226419)

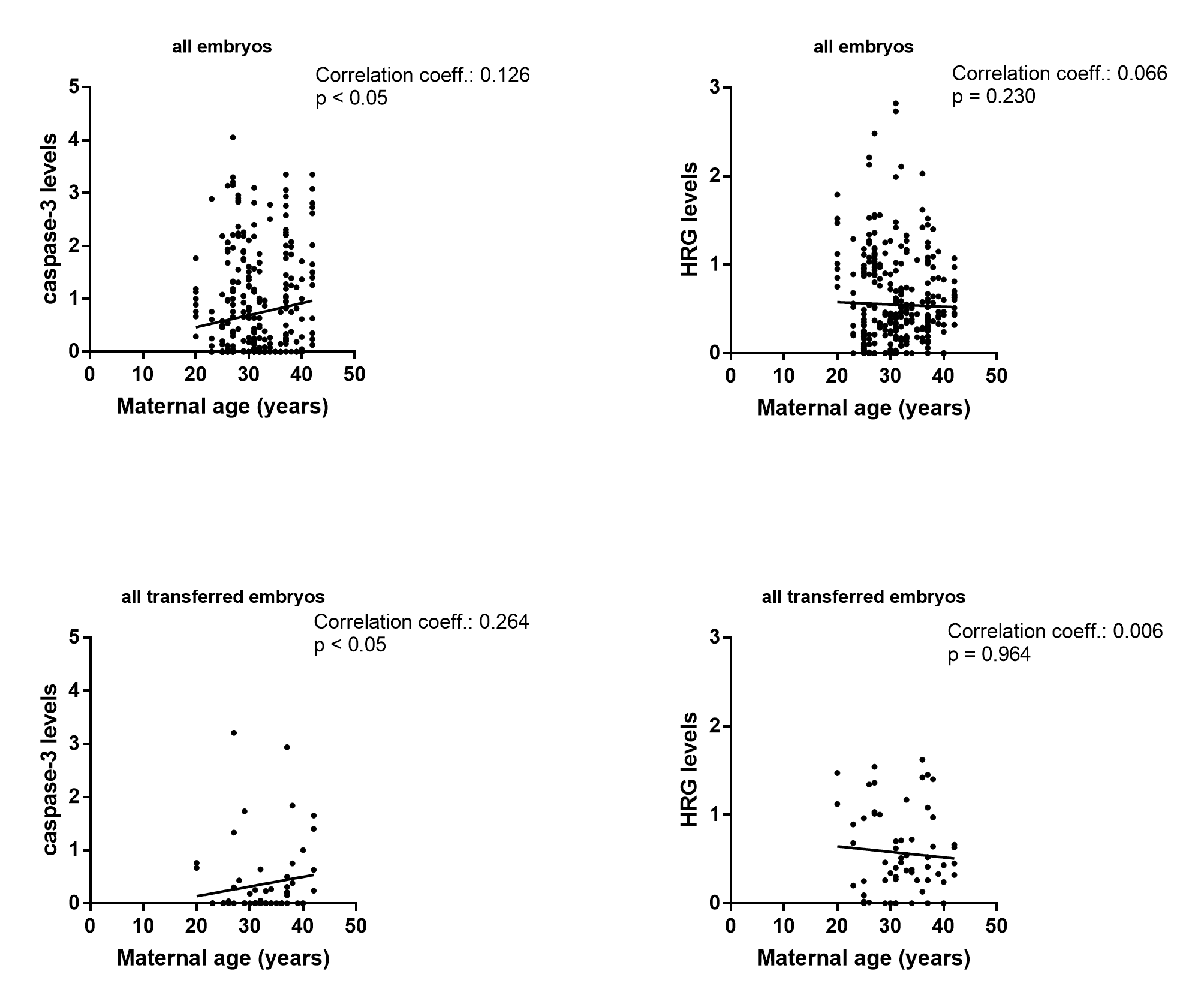

Supplement: S1 Fig — The figure shows the correlation between age vs. caspase-3 and HRG levels in the secretome from all cultured embryos (n = 334) and from all transferred embryos (n = 63). The correlation coefficient and significance were calculated using Spearman’s rank correlation. (TIF) [file pone.0226419.s001.tif]

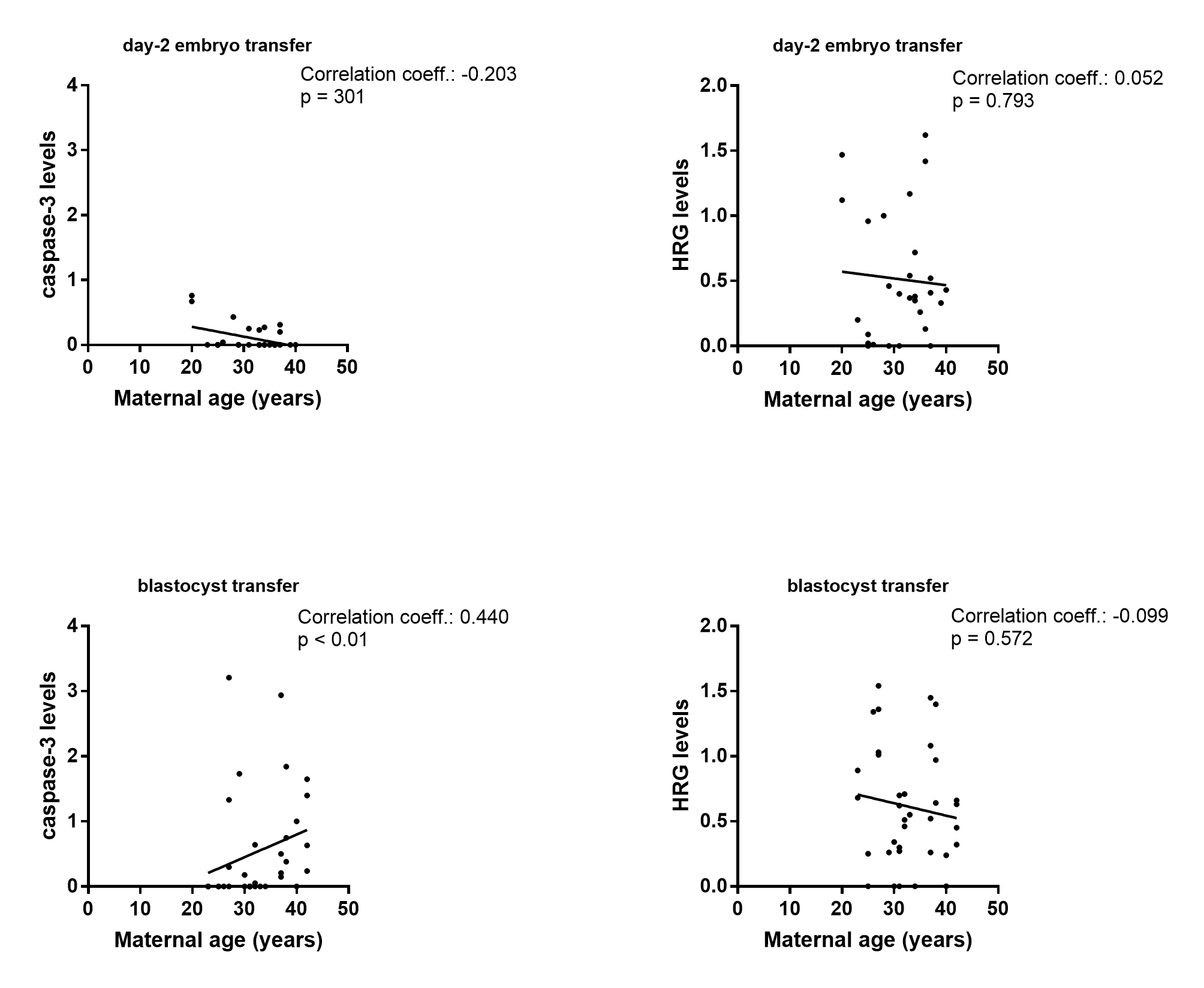

Supplement: S2 Fig — The figure shows the correlation between age vs. caspase-3 and HRG levels in the secretome from transferred day-2 cultured embryos (n = 28) and blastocysts (n = 35). The correlation coefficient and significance were calculated using Spearman’s rank correlation. (TIF) [file pone.0226419.s002.tif]
