# Supplementary material for: Levels of caspase-3 and histidine-rich glycoprotein in the embryo secretome as biomarkers of good-quality day-2 embryos and high-quality blastocysts
Source: PLoS One. 2019 Dec 19;14(12):e0226419. doi: 10.1371/journal.pone.0226419 (PMC6922338; doi:10.1371/journal.pone.0226419)
Supplement: S1 Table — (DOCX) [file pone.0226419.s003.docx]

**S1 Table. Caspase-3 and HRG levels in secretomes from transferred day-2 cultured embryos.**

|  |  | **Not pregnant** | **Pregnant** | **p-value** |
| --- | --- | --- | --- | --- |
| **Caspase-3** | **GT-L (n = 9/6)** | 0.20 (0.00–0.76) | 0.00 (0.00–0.00)* | 0.017* |
|  | **SAGE-1 (n = 6/4)** | 0.00 (0.00–0.25) | 0.00 (0.00–0.27) | 1.000 |
| **HRG** | **GT-L (n = 9/6)** | 0.37 (0.00–1.47) | 0.25 (0.00–0.96) | 0.636 |
|  | **SAGE-1 (n = 6/4)** | 0.78 (0.00–1.62) | - 1. (0.13–0.72) | 0.394 |

The embryos were grouped according to the culture medium used. The table shows the relative levels of caspase-3 and HRG in the embryo secretome. Data are presented as medians (minimum–maximum).

*p ≤ 0.05 compared with not pregnant; Mann–Whitney *U* test.
