# Supplementary material for: Levels of caspase-3 and histidine-rich glycoprotein in the embryo secretome as biomarkers of good-quality day-2 embryos and high-quality blastocysts
Source: PLoS One. 2019 Dec 19;14(12):e0226419. doi: 10.1371/journal.pone.0226419 (PMC6922338; doi:10.1371/journal.pone.0226419)
Supplement: S2 Table — (DOCX) [file pone.0226419.s004.docx]

**S2 Table. Caspase-3 and HRG levels in secretomes from embryos cultured to blastocyst stage.**

|  |  | **Arrested** | **LQB** | **HQB** | **p-value**  **LQB *vs.* arrested** | **p-value**  **HQB *vs.* arrested** | **p-value**  **HQB *vs.* LQB** |
| --- | --- | --- | --- | --- | --- | --- | --- |
| **Caspase-3** | **GT-L (n = 47/12/13)** | 0.96 (0.00–3.14) | 0.78 (0.00–3.35) | 0.00 (0.00–2.94)* | 0.599 | 0.019* | 0.031* |
|  | **SAGE-1 (n = 98/7/25)** | 0.62 (0.00–3.35) | 0.24 (0.00–0.76) | 0.24 (0.00–3.15) | 0.452 | 0.148 | 0.643 |
| **HRG** | **GT-L (n = 47/12/13)** | 0.20 (0.00–1.99) | 0.34 (0.00–1.42) | 0.23 (0.00–1.18) | 0.392 | 0.772 | 0.361 |
|  | **SAGE-1 (n = 98/7/25)** | 0.55 (0.00–2.48) | 0.45 (0.14–0.94) | 1.01 (0.00–1.45)* | 0.375 | 0.011* | 0.040* |

Embryos reaching blastocyst stage were divided into two groups depending on quality: low-quality blastocysts (LQB) and high-quality blastocysts (HQB). The embryos were further divided into groups depending on the culture medium used. The table shows the relative levels of caspase-3 and HRG in the embryo secretome. Data are presented as medians (minimum–maximum).

*p ≤ 0.05 compared with arrested embryos and LQB; Mann–Whitney *U* test
