# Supplementary material for: Levels of caspase-3 and histidine-rich glycoprotein in the embryo secretome as biomarkers of good-quality day-2 embryos and high-quality blastocysts
Source: PLoS One. 2019 Dec 19;14(12):e0226419. doi: 10.1371/journal.pone.0226419 (PMC6922338; doi:10.1371/journal.pone.0226419)
Supplement: S3 Table — (DOCX) [file pone.0226419.s005.docx]

**S3 Table. Caspase-3 and HRG levels in the secretomes from transferred blastocysts.**

|  |  | **Not pregnant** | **Pregnant** | **p-value** |
| --- | --- | --- | --- | --- |
| **Caspase-3** | **GT-L (n = 3/3)** | 0.00 (0.00–2.94) | 0.00 (0.00–0.00) | 0.317 |
|  | **SAGE-1 (n = 16/13)** | 0.22 (0.00–1.65) | 0.18 (0.00–3.21) | 0.752 |
| **HRG** | **GT-L (n = 3/3)** | 0.26 (0.00–0.27) | 0.25 (0.00–0.70) | 1.000 |
|  | **SAGE-1 (n = 16/13)** | 0.64 (0.00–1.45) | 0.62 (0.00–1.54) | 0.895 |

The embryos were grouped according to the culture medium used. Data are presented as medians (minimum–maximum). The Mann–Whitney *U* test was used for statistical analysis.
